# The comparison of colpocleisis and sacrospinous ligament fixation surgeries for treatment of vaginal vault prolapse: an observational study

**DOI:** 10.12669/pjms.41.12.12333

**Published:** 2025-12

**Authors:** Elcin Islek Secen, Emre Erdem Tas

**Affiliations:** 1 Elcin Islek Secen, MD., Ankara Yıdırım Beyazıt University, Faculty of Medicine, Department of Obstetrics and Gynecology, Ankara, Turkey; 2 Emre Erdem Tas, MD, Ankara Yıdırım Beyazıt University, Faculty of Medicine, Department of Obstetrics and Gynecology, Ankara, Turkey

**Keywords:** Colpocleisis, Sacrospinous ligament fixation, Vaginal vault prolapse

## Abstract

**Objective:**

This study aimed to compare the surgical outcomes, as well as the intraoperative and postoperative complications, of colpocleisis and sacrospinous ligament fixation (SSLF) in the management of vaginal vault prolapse (VVP) among post-hysterectomy patients.

**Methods:**

A retrospective, observational study was conducted on 49 patients who underwent surgery for VVP at Ankara Bilkent City Hospital between January 2020 and December 2024. Of the 49 patients, 26 (53.1%) underwent colpocleisis surgery and 23 (46.9%) underwent SSLF surgery. Data collected included patient demographics, preoperative and postoperative hemoglobin levels, operation time, hospital stay, and postoperative complications. The study also assessed treatment success and recurrence rates.

**Results:**

The mean age of the patients was significantly higher in the colpocleisis group than the SSLF group (71.5 years *vs*. 59.9 years, p=0.01). There were no significant differences in hemoglobin drop levels, previous hysterectomy indications and types, or operation times between the two groups (p≥0.05). The mean hospital stay was significantly longer in the colpocleisis group than in the SSLF group (3.2 days *vs*. 1.6 days, p=0.02). Kaplan–Meier analysis revealed no significant difference in VVP recurrence requiring reoperation between the colpocleisis and SSLF groups (3.8% vs. 13.1%, respectively; p = 0.13).

**Conclusion:**

SSLF and colpocleisis have demonstrated high efficacy in the treatment of vaginal vault prolapse. These surgical approaches are particularly appropriate for elderly patients with significant comorbidities, as they are associated with low complication rates, shorter operative times, and reduced recurrence rates.

## INTRODUCTION

Pelvic organ prolapse (POP) is defined as the descent of one or more aspects of the vagina and uterus: the anterior vaginal wall, posterior vaginal wall, uterus (cervix), or vaginal vault.^1^ POP is a common problem that increases in frequency with age and impairs quality of life. However, examinations reveal some degree of prolapse in 40-60% of parous women.^2^,^3^ Post-hysterectomy vaginal vault prolapse (VVP), which can be graded using the Pelvic Organ Prolapse Quantification System classification, is defined as the descent of the apex of the vagina (vaginal vault or cuff scar after hysterectomy) due to the loss of apical support of the vagina after hysterectomy.^4^ Hysterectomy, performed for obstetric, benign, or malignant indications, ranks among the most common gynecological procedures, with an estimated incidence of 351 per 100,000 persons a year.^5^ The incidence of VVP is estimated to be 36 per 10,000 women.^6^ The risk of apical prolapse after hysterectomy is higher than that in non-hysterectomized controls, although age, parity, obesity, and chronic lung disease are other risk factors for the development of POP.^3^,^7^

VVP is often associated with other compartment defects (cystocele, rectocele, or enterocele), making it difficult to treat.^8^ Due to the significant contribution of the apex to vaginal support, anterior and posterior vaginal repairs may fail if the apex is not adequately supported.^2^,^8^,^9^ While some studies advocate surgical intervention due to increased life expectancy, others recommend first-line conservative treatment to avoid surgical risks.^10^ The choice of treatment is determined by the severity of the prolapse, the patient’s current health status and comorbidities. Prolapse surgery can be performed using an obliterative or a reconstructive approach. Colpocleisis, an obliterative vaginal procedure, demonstrates high success rates and low perioperative morbidity, making it a preferred option for older women who are unable to tolerate extensive surgery or are not sexually active. It has a short anesthesia requirement, short operative time, and high surgical success rate.^11^-^13^

Reconstructive methods can be performed using the abdominal and vaginal approaches. Abdominal procedures include sacrocolpopexy and uterosacral advancement operations, which can be performed via laparoscopy and laparotomy. Conversely, sacrospinous ligament fixation (SSLF), a vaginal approach, is an effective technique for correcting prolapse and vaginal restoration by fixing the vaginal cuff to the sacrospinous ligament. SSLF is a commonly preferred technique by surgeons because of reports that advocate that it causes fewer complications, less postoperative pain, and shorter hospital stays than transabdominal techniques.^14^,^15^ However, complications such as urinary incontinence, nerve damage, recurrence, and problems involving sexual intercourse may occur after POP surgeries.^11^-^16^

The literature still has gaps regarding the short and long-term outcomes of surgical interventions for the treatment of VVP. The aim of this study was to compare the surgical outcomes of colpocleisis and SSLF surgeries in the treatment of VVP in post-hysterectomy patients.

## METHODOLOGY

This study retrospectively reviewed the medical records of 49 patients who underwent surgery for VVP at Ankara Bilkent City Hospital, Ankara, Turkey, between January 2020 and December 2024.

### Ethical Approval:

It was obtained from the Institutional Clinical Research and Ethics Committee (1-25-946; date April 20, 2023), and written informed consent was obtained from all participants. This study was conducted in accordance with the 1964 Declaration of Helsinki and its subsequent amendments and was reported in accordance with the Strengthening the Reporting of Observational Studies in Epidemiology (STROBE) statement.

Data regarding patient demographic characteristics (age, gravidity, and parity), body mass index (BMI, kg/m^2^), medical and surgical history, preoperative serum hemoglobin levels (g/dL), operation type (i.e., colpocleisis *vs*. sacrospinous ligament fixation), duration of operation (min), postoperative serum hemoglobin drop level (g/dL), duration of hospital stay (days), and postoperative treatment success (i.e., success *vs*. recurrence) were obtained from the hospital medical database. A total of 49 patients were included in the study, as complete data were available for all cases and each patient was followed up for at least 12 months postoperatively.

In all cases, the surgical approach was determined following evaluation by our clinic’s preoperative council, taking into account patient-specific factors such as age, sexual activity, comorbidities, and functional status. At our clinic, colpocleisis is preferred—with informed consent—for elderly, sexually inactive patients with significant comorbidities in cases of VVP, while reconstructive procedures such as SSLF are favored for sexually active patients.

All cases of vaginal vault prolapse included in the study were classified according to the Pelvic Organ Prolapse Quantification (POP-Q) system. In our clinic, standardized surgical techniques are employed for both procedures. The SSLF operation is performed vaginally, with the right sacrospinous ligament anchored to the vaginal cuff using permanent monofilament sutures. In cases where anterior and/or posterior vaginal wall prolapse is also present, concomitant repairs are performed as required. On the other hand, colpocleisis involved the excision of most of the vaginal epithelium, extending posteriorly from within the hymenal ring and anteriorly to approximately 1.0–2.0 cm from the external urethral meatus.

Treatment recurrence was defined as the anatomical reappearance of apical prolapse during follow-up, specifically a POP-Q stage ≥ II at the apical compartment. Immediate reoperation performed within six weeks postoperatively was not considered recurrence; however, reoperations beyond this period were classified as recurrence. Treatment failure was defined as the inability to achieve adequate apical support post-treatment, indicated by anatomical recurrence according to the above criteria or persistence of clinical symptoms.

### Statistical analysis:

Descriptive parameters were expressed as mean ± standard deviation for continuous variables and as numbers and percentages for categorical variables. The groups (colpocleisis *vs*. SSLF) were compared using independent sample t-tests and chi-squared tests. Kaplan-Meier curves were generated to visualize differences in VVP recurrence over time between the surgical approaches. Statistical analyses were performed using the Statistical Package for the Social Sciences for Windows, version 21.0 (IBM, SPSS Corp.; Armonk, NY, USA). Statistical significance was set at P<0.05. Power analysis of the study was conducted using G*Power3.0 software (Germany), revealing 80.7% power using Type-I error (α)=0.05, effect size=0.5, and a two-sided t-test.

## RESULTS

**Fig.1:**
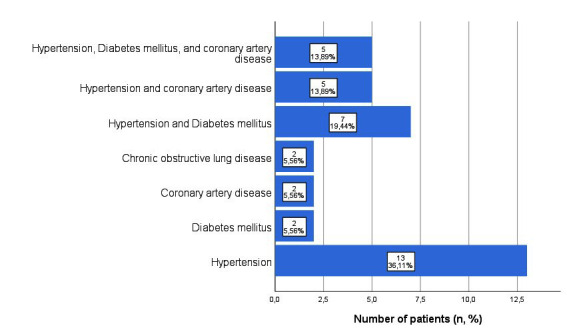
Concomitant medical disease in patients with vaginal vault prolapse.

The means of all patients age, gravidity, parity and BMI were 66.0±10.3 years, 4.6±2.5, 3.6±1.8, and 24.8±3.3 kg/m^2^ respectively. Except for three patients in the SSLF group, all patients were in menopause. Of the 49 patients, 36 (73.5%) had concomitant medical diseases (Fig.1). The mean time interval between hysterectomy and vaginal vault prolapse was 10.3±9.5 years. Approximately half of the patients had undergone hysterectomy for POP and the other half for other benign gynecological diseases, such as abnormal uterine bleeding and leiomyoma. The previous hysterectomy types included total abdominal hysterectomy (n=26, 53.1%) and vaginal hysterectomy (n=23, 46.9%). All patients had stage four apical VVP according to the POP-Q classification system. Concomitant urge-type urinary incontinence was present in four of the 49 patients (8.2%).

**Fig.2:**
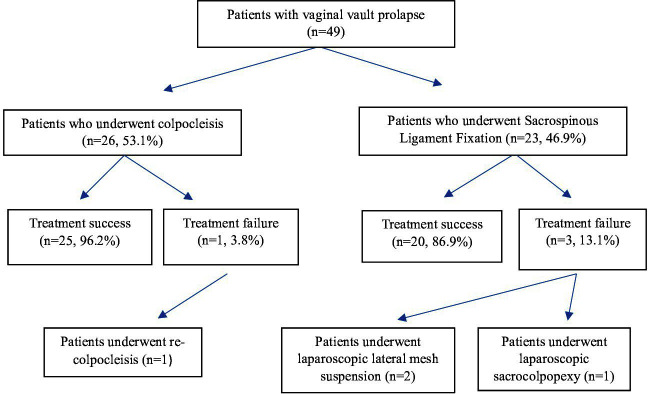
The flow-chart of the study population.

Of the 49 patients, colpocleisis was performed in 26 (53.1%) patients, and SSLF was performed in 23 (46.9%) patients. The mean hospital stay was 2.4 ± 2.3 days. The mean follow-up duration of all patients was 25.4 ± 11.1 months. Among them, the mean follow-up was 27.8 ± 13.8 months in patients who underwent colpocleisis and 22.6 ± 6.0 months in those who underwent SSLF. The difference between the groups was not statistically significant (p=0.10). In the postoperative period, surgical treatment recurrence occurred in four patients (8.1%). There was no failure before the 6th postoperative week. Of these, two underwent laparoscopic lateral mesh suspension, one underwent laparoscopic sacrocolpopexy, and one underwent colpocleisis (Fig.2).

The age and gravidity was significantly higher in the colpocleisis group than the SSLF group (71.5 years *vs*. 59.9 years, p=0.01; 5.4 *vs*. 3.7, p=0.01, respectively). However, there was no significant difference between the groups in terms of gravidity, BMI, concomitant medical disease ratio, time interval between hysterectomy and prolapse (years), and previous hysterectomy indications and types (p≥0.05) (Table-I).

**Table-I:** Clinical characteristics of the colpocleisis and sacrospinous ligament fixation groups.

Characteristics	Colpocleisis group (n=26, 53.1%)	Sacrospinous ligament fixation group (n=23, 46.9%)	P*
Age (years)	71.5±7.2	59.8±9.7	0.01
Gravidity	5.4±2.8	3.7±1.9	0.01
Parity	4.1±1.9	3.1±1.7	0.06
Body mass index (kg/m^2^)	24.0±3.2	25.7±3.3	0.08
***Concomitant medical disease***			
Yes	20 (40.8)	16 (32.7)	0.74
No	6 (12.2)	7 (14.3)	
Time interval between hysterectomy and prolapse (years)	12.2±10.0	8.3±8.1	0.15
***Previous hysterectomy indication***			
Pelvic organ prolapse	16 (32.7)	8 (16.3)	15 (30.6)
Other benign gynecological diseases	10 (20.4)	0.08
*Previous hysterectomy type*			
Total abdominal hysterectomy	12 (24.5)	14 (28.6)	0.39
Vaginal hysterectomy	14 (28.6)	9 (18.4)	
Preoperative serum hemoglobin level (g/dL)	12.5±1.2	12.9±1.2	0.28
Hemoglobin drop level (g/dL)	0.9±0.7	1.2±0.5	0.24
Operation time (min)	74.0±20.2	71.7±20.6	0.69
Hospital stay (day)	3.2±2.9	1.6±1.1	0.02

The data is presented as the mean ± standard deviation or n (%).

* Independent sample t-tests and chi-squared tests were used to compare groups.

**Fig.3:**
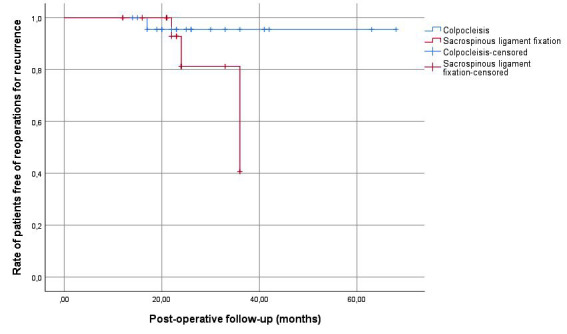
Kaplan-Meier curves for apical vault prolapse recurrence according to the type of surgery (colpocleisis *vs*. sacrospinous ligament fixation).

The colpocleisis and SSLF groups did not differ in terms of preoperative serum hemoglobin levels, hemoglobin drop levels, or mean operation time (p≥0.05). However, the mean hospital stay was significantly longer in the colpocleisis group than in the SSLF group (3.2 days *vs*. 1.6 days, p=0.02), (Table-I). According to the Kaplan–Meier analysis, the rate of VVP recurrence requiring reoperation was comparable between the colpocleisis and SSLF groups (3.8% vs. 13.1%, respectively; p = 0.13) (Fig.3).

## DISCUSSION

With the increase in elderly population, the prevalence of pelvic floor disorders, including POP, is expected to rise substantially. Accordingly, there has been an anticipated increase in POP surgeries.^17^,^18^ However, with numerous surgical techniques available, establishing a standardized and optimal treatment protocol for VVP remains challenging due to the heterogeneity of patient populations and surgical options. While various studies have explored the advantages and disadvantages of reconstructive versus obliterative surgeries for POP^2^,^11^,^16^, direct comparisons specifically between colpocleisis and SSLF for VVP are limited. In our study, 49 patients who underwent SSLF and colpocleisis were evaluated, and no significant differences were found in their demographic data except for age and gravidity. Reoperation was required in 13.8% of patients who underwent SSLF and 3.8% of those who underwent colpocleisis. Although the recurrence rate was higher in SSLF, this rate was not found to be statistically significant, and both methods were shown to be effective and reliable for VVP.

Colpocleisis is generally preferred as a surgical treatment option for pelvic organ prolapse in elderly women. Multiple studies have reported that this procedure is most commonly performed in patients aged 70 years and older.^13^,^19^,^20^ This finding is consistent with the results of our study, where the mean age of patients who underwent colpocleisis was 71.5 years, while the mean age of patients in the SSLF group was significantly younger at 59.8 years. The age difference between the two groups was statistically significant. This outcome aligns with the literature, as colpocleisis is typically an obliterative surgical procedure reserved for women who are no longer sexually active and do not intend to resume vaginal intercourse.

An important factor in POP is the history of hysterectomy. Studies, including the Oxford Family Planning Association study, show that the risk of prolapse increases over time after hysterectomy—from 1% at three years to 5% at fifteen years. Women who had hysterectomy due to prolapse have a 5.5-fold higher risk of recurrent prolapse compared to other indications.^21^ In our study, the mean interval between hysterectomy and vaginal vault prolapse was 10.3 years, with no significant difference between the colpocleisis and SSLF groups. Additionally, initial hysterectomy indications and types were similar between groups. This similarity strengthens our study’s reliability by minimizing bias in recurrence risk assessment and surgical outcomes, suggesting that the results reflect the true efficacy of SSLF and colpocleisis without influence from prior hysterectomy factors.

In the study conducted by Agacayak et al., which compared SSLF and colpocleisis, postoperative hospital stay and operation time were found to be significantly longer in the SSLF group compared to the colpocleisis group.^16^ In contrast, our study did not reveal a significant difference in operation time between the two groups, although hospital stay was significantly longer in the colpocleisis group. In the study by Agacayak et al., the patient age groups were comparable to those in our study; however, 86% of the patients in their SSLF group underwent an additional hysterectomy during the operation. Furthermore, the longer hospital stay observed in the colpocleisis group may be attributed to the need for extended follow-up due to older age and associated comorbidities. Additionally, all patients had post-hysterectomy VVP, which may explain the comparable operation times between groups.

Studies suggest colpocleisis is preferred for elderly or comorbid patients who may not tolerate prolonged surgery, due to shorter operation times and lower perioperative morbidity.^12^,^13^,^19^,^20^ No previous study has directly compared SSLF and colpocleisis in VVP, though reconstructive surgery is generally associated with higher complication risks than POP surgery.^2^,^11^ These complications mostly occur in abdominal procedures. Solomon et al. reported 2.8% postoperative urinary retention and 0.7% blood transfusion due to bladder or urethral injuries after SSLF, with no nerve damage.^22^ Kansaria et al. found complications in 29.7% of SSLF patients for VVP, mainly urinary tract infections (16.7%) and minor issues like urinary retention and hip pain.^14^ Our study found no difference in operation time or hemoglobin drop between groups, and no serious intra- or postoperative complications were observed.

A systematic review by Morgan et al.^23^ analyzing 17 studies reported an anatomic failure rate of 10.3% using, stage 2 prolapse as the criterion for failure. However, these studies included both uterine and vaginal vault prolapse cases. An observational study by Brunes et al.^24^ found that 18.1% of patients undergoing SSLF without graft required reoperation, compared to 7.8% with graft use. Studies have shown colpocleisis recurrence rates below 5% with high success. In our study, 13% of SSLF patients required reoperation, with two undergoing laparoscopic lateral mesh suspension and one laparoscopic sacrocolpopexy. Only one colpocleisis patient (3.8%) required reoperation. While obliterative surgeries generally show lower recurrence rates than reconstructive ones, we found no significant difference between SSLF and colpocleisis. This discrepancy may be due to heterogeneous patient groups in other studies combining uterine and vaginal vault prolapse, and obliterative and reconstructive surgeries.

### Strength of the study:

It has its focus on a homogeneous patient population—post-hysterectomy women with vaginal vault prolapse—which reduces variability and enhances the reliability of the comparison between SSLF and colpocleisis. Importantly, the absence of significant differences in operative time, major complications, and recurrence rates suggests that both procedures are equally effective and safe for this group. These findings imply that the choice between SSLF and colpocleisis in vaginal vault prolapse should be based on patient-specific factors such as sexual activity, overall health, and surgical preference, rather than differences in clinical outcomes.

### Limitations:

It primarily focused on anatomical outcomes and did not evaluate patient-reported functional outcomes or quality of life measures. Subjective aspects such as sexual function and patient satisfaction were not assessed with standardized questionnaires, limiting the understanding of the overall treatment impact. Additionally, the relatively small sample size and retrospective design may reduce the generalizability of the findings.

## CONCLUSION

Surgical management of VVP should be individualized based on patient factors such as health status, comorbidities, age, and sexual activity. Although recurrence was higher in the SSLF group, this difference was not statistically significant. Both SSLF and colpocleisis proved effective with similar operative times, complication rates, and recurrence rates. Given the benefits of vaginal approaches, including fewer complications, shorter surgeries, and less bleeding, vaginal surgery is preferable—especially for patients with comorbidities or limited surgical tolerance. Ultimately, the choice between SSLF and colpocleisis should be guided by patient-specific factors—such as age, comorbidities, and sexual activity—rather than presumed differences in surgical efficacy.

### Recommendations:

Future prospective studies with larger cohorts are needed to validate and expand upon these results.
